# A preliminary and qualitative study of resource ratio theory to nitrifying lab-scale bioreactors

**DOI:** 10.1111/1751-7915.12284

**Published:** 2015-04-15

**Authors:** Micol Bellucci, Irina D Ofiţeru, Luciano Beneduce, David W Graham, Ian M Head, Thomas P Curtis

**Affiliations:** 1 School of Civil Engineering and Geosciences, Newcastle UniversityNewcastle upon Tyne, NE1 7RU, UK; 2 Dipartimento di Scienze Agrarie, Alimentari ed Ambientali, Università di Foggiavia Napoli 25, Foggia, 71122, Italy; 3 School of Chemical Engineering and Advanced Materials, Merz Court, Newcastle UniversityNewcastle upon Tyne, NE1 7RU, UK; 4 Chemical Engineering Department, University Politehnica of BucharestPolizu 1-7, Bucharest, 011061, Romania

## Abstract

The incorporation of microbial diversity in design would ideally require predictive theory that would relate operational parameters to the numbers and distribution of taxa. Resource ratio-theory (RRT) might be one such theory. Based on Monod kinetics, it explains diversity in function of resource-ratio and richness. However, to be usable in biological engineered system, the growth parameters of all the bacteria under consideration and the resource supply and diffusion parameters for all the relevant nutrients should be determined. This is challenging, but plausible, at least for low diversity groups with simple resource requirements like the ammonia oxidizing bacteria (AOB). One of the major successes of RRT was its ability to explain the ‘paradox of enrichment’ which states that diversity first increases and then decreases with resource richness. Here, we demonstrate that this pattern can be seen in lab-scale-activated sludge reactors and parallel simulations that incorporate the principles of RRT in a floc-based system. High and low ammonia and oxygen were supplied to continuous flow bioreactors with resource conditions correlating with the composition and diversity of resident AOB communities based on AOB 16S rDNA clone libraries. Neither the experimental work nor the simulations are definitive proof for the application of RRT in this context. However, it is sufficient evidence that such approach might work and justify a more rigorous investigation.

## Introduction

There is growing consensus on the key role of species richness of ecosystem function (Hooper *et al*., 2005; Cardinale *et al*., 2006). Empirical and theoretical studies in ecology suggest that elevated species richness improves ecosystem functional stability, especially system resilience to perturbations (Pimm, 1984; Tilman and Downing, 1994; Tilman *et al*., 2001). In principle, more diverse systems have a greater pool of physiological and genetic traits, which provide them the capacity to change and sustain function under varying environmental conditions.

This observation is of particular relevance to the engineering of open biological systems. Activated sludge and biofilm systems with high diversity have been shown to sustain functionality aftershock loading of mercury for example (von Canstein *et al*., 2002; Saikaly and Oerther, 2011). Similarly, studies on nitrifying systems suggested that a more diverse ammonia-oxidizing microbial community is more resistant to operational variability and can enhance the reliability of the process (Daims *et al*., 2001; Egli *et al*., 2003; Rowan *et al*., 2003). However, the exact mechanism underlying the beneficial effect of diversity is still uncertain. Wittebolle and colleagues (2009) argued that the initial evenness of the community rather than species richness *per se* is the key factor for the functional stability of the system. Nevertheless, it seems likely that increasing microbial diversity will do no harm and may improve stability.

The rational engineering of microbial diversity would be possible if one could incorporate theoretical ecology into process design (Curtis *et al*., 2003), which is both intrinsically fascinating and deeply practical. However, the body of available theory is modest and largely untested.

One plausible approach is Tilman's resource-ratio theory (RRT; Tilman, 1982). Tilman used the Monod kinetics, familiar to engineers, to explain the variation of diversity with the quantity and ratio of resources supplied. According to the RRT, individual populations consume resources and increase in size until one resource becomes limiting. In heterogeneous environments, the theory predicts that (i) high species diversity and evenness occur when low to intermediate amount of resources are supplied, while resource enrichment leads to decreased species richness and evenness, (ii) high variations of the two resources lead to increased species richness and (iii) the resource supply ratio determines which species are dominant. In addition, the RRT provides one of the most elegant theoretical explanations for the ‘paradox of enrichment’ noted by Rosenzweig (1971). In essence this paradox is that increasing the amount of resource causes and initial increase and then a substantial decrease of the number of taxa within a system, leading to a destabilization of the ecosystem. Resource ratio-theory has been used to explain the biological community structure in terrestrial and aquatic plants, plankton, dental plaque and microbial populations in hydrocarbon-contaminated systems (Tilman, 1982; Smith, 1983; 1993; Smith *et al*., 1998). A critique of these studies has suggested that the results of most of these tests are inconclusive (Miller *et al*., 2005).

In principle, engineered biological processes are ideal systems for testing both the principles of the RRT and the paradox of enrichment because pollutants (growth limiting resources) supplied to the microbial community can be easily controlled. One important case where RRT might be used is nitrification in activated sludge treatment plants. Nitrification is a critical step in many wastewater treatment plants (WWTPs) including nitrogen removal process. During a conventional nitrification process, the ammonia oxidizing bacteria (AOB) oxidize the ammonia to nitrite, which is then converted to nitrate by nitrite oxidizing bacteria (NOB). However, nitrification often fails unexpectedly. If the predictions of RRT are correct, low oxygen and ammonia inputs should select for a more diverse AOB community. This, in turn, should increase the reliability of the biological ammonia oxidation process. Hence, reducing oxygen levels and prospective energy consumption for aeration might have the simultaneous benefit of reducing the risk of nitrification failure. A truly rigorous test of RRT in a nitrifying wastewater treatment plant is a daunting prospect. It would require us to know the maximum specific growth rates and the half saturation coefficients for ammonia and oxygen of dozens of taxa, as well as the parameters required to describe the diffusion and consumption of ammonia and oxygen across the population of flocs.

Our goal was more limited, to qualitatively evaluate the ability of RRT to describe variation of the AOB diversity in lab-scale nitrifying bioreactors. The experimental studies were complemented by a parallel simulation that incorporates the principles of RRT in a floc-based system where the resource ratio is controlled by diffusion into the biomass. High and low ammonia and oxygen supplies (2 × 2 design) were provided to different units with resource conditions and correlated with the composition and diversity of resident AOB communities based on AOB 16S ribosomal ribonucleic acid (rRNA) clone libraries. The ‘paradox of enrichment’ was qualitatively demonstrated both in lab reactors and in the parallel computational simulations.

## Results

### Reactor's performance

Four reactors were set up (Table1) and designate (R1) had low nitrogen and high oxygen (L_N_H_O_), R2, low nitrogen and low oxygen (L_N_L_O_), R3 high nitrogen and high oxygen (H_N_H_O_) and R4 high nitrogen and low oxygen (H_N_L_O_).

**Table 1:** Main operational parameters of the bioreactors

	R1-L_N_H_O_	R2-L_N_L_O_	R3-H_N_H_O_	R4-H_N_L_O_
COD _infl_ (mg/l)	528.2 ± 32.5	528.2 ± 32.5	528.2 ± 32.5	528.2 ± 32.5
NH_4_^+^-N _infl_ (mg/l)	24.2 ± 5.5	24.2 ± 5.5	280 ± 17	280 ± 17
Oxygen supplied (%)	21	2	21	2
Air flow rate (l/min)	0.2	0.2	0.2	0.2
DO (mg/l)	3.79 ± 0.49	0.26 ± 0.2	3.37 ± 1.43	0.18 ± 0.24
Volume (l)	3	3	3	3
SRT (days)	5	5	5	5
Temperature (°C)	20 ± 1.6	20 ± 1.6	20 ± 1.6	20 ± 1.6

**Fig 1:**
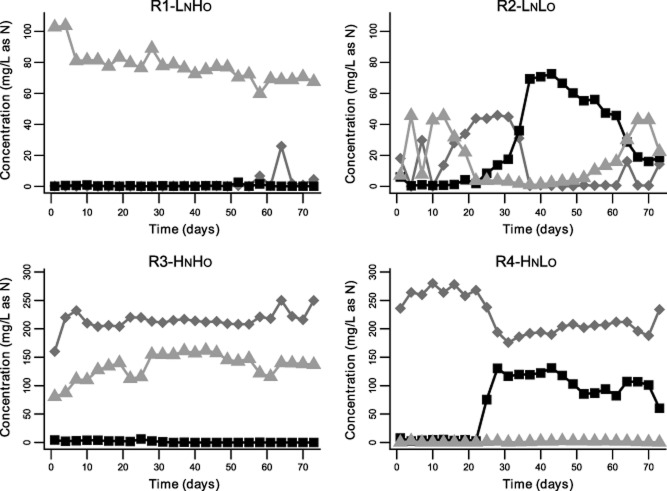
Concentration of ammonia (◆), nitrite (■) and nitrate (▲) detected in the reactors over time.

Ammonia oxidation was achieved in all systems, although time scales and rates varied (Fig. 1). Complete ammonium removal occurred within 4 and 37 days from inoculation in R1-L_N_H_O_ and R2-L_N_L_O_, respectively. In R3-H_N_H_O_, the ammonia consumption was 22.3% ± 4.4% of the total ammonium concentration of the influent, while in R4-H_N_L_O_ an average of 29.1% ± 4.4% of ammonium was removed after 28 days of operation. Free ammonia and free nitrous acid concentration were almost negligible in R1-L_N_H_O_, while they varied over time in the other bioreactors (Fig. S2 and SI).

Dissolved oxygen (DO) concentrations varied in the systems according to the mode of aeration and ammonia levels in the influent [analysis of variance (ANOVA), *n* = 25, *P-*value < 0.05]. The DO concentration averaged 3.58 ± 1.08 mg/l in R1-L_N_H_O_ and R3-H_N_H_O_, and 0.22 ± 0.22 mg/l in R2-L_N_L_O_ and R4-H_N_L_O_. The different resources supplied affected also the pH (ANOVA, *n* = 25, *P-*value < 0.05) that was 8 ± 0.4 in R1-L_N_H_O_, 8.05 ± 1.54 in R2-L_N_L_O_ and 7.5 ± 0.72 in R4-H_N_L_O_; a drastic decrease of pH to 6.08 ± 0.86 was observed in R3-H_N_H_O_. The chemical oxygen demand (COD) removal rates were very high during the entire experiment. In R1-L_N_H_O_ and R3-H_N_H_O_, the COD removal stabilized at an average level of 93.7% ± 2% after 4 days of operation. In the low DO reactors (R2-L_N_L_O_ and R4-H_N_L_O_), the COD removal was higher than 89.5% ± 4.9% until the ammonia oxidation activity started. Then, it decreased to 79.9% ± 6.3% and 71.6% ± 5.1% in R2-L_N_L_O_ and R4-H_N_L_O_, respectively. The volatile suspended solid (VSS) concentration stabilized 10 days after inoculation, averaging 257 ± 47 mg/l in all configurations.

### Abundance and dynamics of the AOB

**Fig 2:**
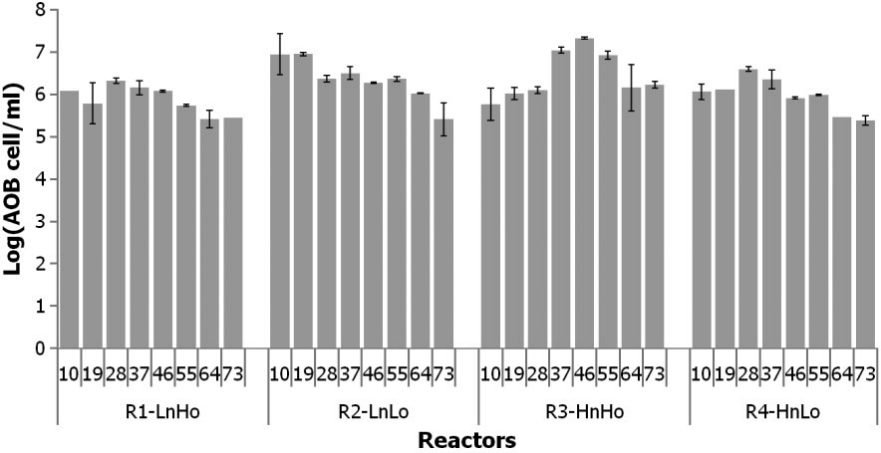
Ammonia oxidizing bacteria abundance in the reactors over time assessed by qPCR. Bars show the mean of duplicate sample, while error bars represent standard deviation.

The abundance of AOB fluctuated over time (Fig. 2). However, there was no significant difference between configurations (ANOVA, *n* = 8, *P-*value = 0.053). Overall, the number of AOB ranged between 2.45 × 10^5^ and 3.96 × 10^6^ cells per ml. We should acknowledge that the AOB quantification could be overestimated as the CTO primers could anneal non-AOB DNA template.

Temporal changes in the denaturant gradient gel electrophoresis (DGGE) profiles of the AOB 16S rRNA gene fragments were used to observe AOB dynamics in all configurations.

**Fig 3:**
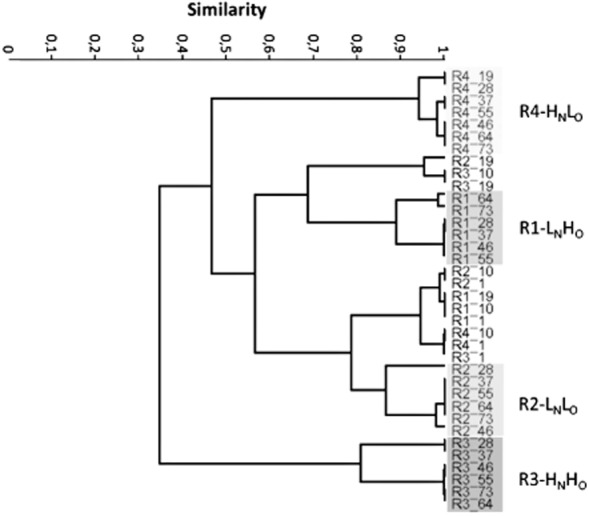
Cluster analysis of the AOB DGGE profiles based on Raup and Crick coefficient. The band patterns of the samples taken 28 days after inoculation from the same unit had grouped together as shown by the shaded boxes.

The DGGE patterns of samples from each reactor were analysed using Raup and Crick diversity indices (S_RC_) before and after 28 days of operation. The results indicate that before day 28, the samples are clustering according to time, while after day 28, they are clustering according to the reactor. This suggests that the AOB community structure in each reactor changed according to the supply of oxygen and ammonia to the systems (Fig. 3).

**Fig 4:**
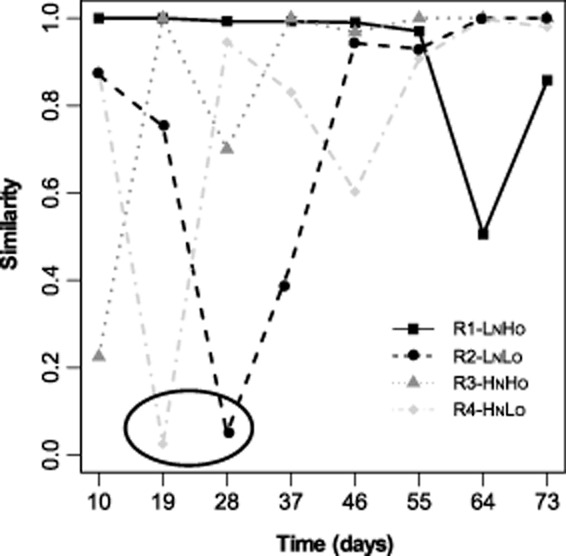
Ammonia oxidizing bacteria community drifts detected by moving window analyses. The similarity between DGGE profiles of samples taken from the same reactor at two consecutive sampling days was calculated based on the Raup and Crick index. The circle highlights that the AOB community changed significantly 19 and 28 days after inoculation in R4-H_N_L_O_ and R2-L_N_L_O_, respectively.

Temporal changes in the AOB community were evident from moving window analysis (Fig. 4). Significant shifts were observed in the two reactors supplied with low oxygen between days 19 and 28 (S_RC_ = 0.025 in R4-H_N_L_O_ and S_RC_ = 0.05 in R2-L_N_L_O_). In R3-H_N_H_O_, the lowest S_RC_ value observed (0.225) was detected between the first and the 10th day of the experiment. The AOB community in R2-L_N_L_O_, R3-H_N_H_O_ and R4-H_N_L_O_ reached an apparent dynamic equilibrium during the last period of the experiment (between days 55 and 73) as the DGGE profiles were significantly similar (S_RC_ > 0.95). In contrast, the AOB community in R1-L_N_H_O_ was quite stable until day 64, when a change occurred; however, the difference between the DGGE profile of days 55 and 64 was modest and no greater than would be expected by random association of bands in the DGGE patterns.

### Composition of the AOB community assessed by 16S rRNA gene clone libraries

**Fig 5:**
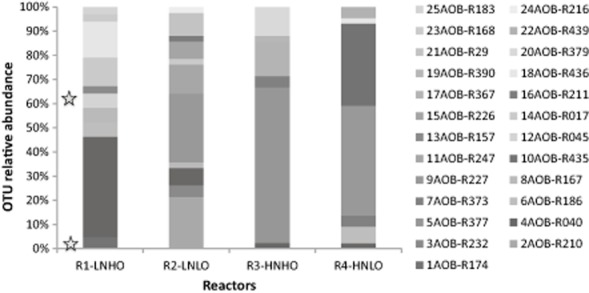
Distribution of AOB 16S rRNA gene clones retrieved from the four reactors after 73 days. The 209 clones were sorted into 25 groups represented by a single OTU (bars in different colours). 12AOB-R045 and 1AOB-R174 (stars) are non-AOB OTUs.

**Fig 6:**
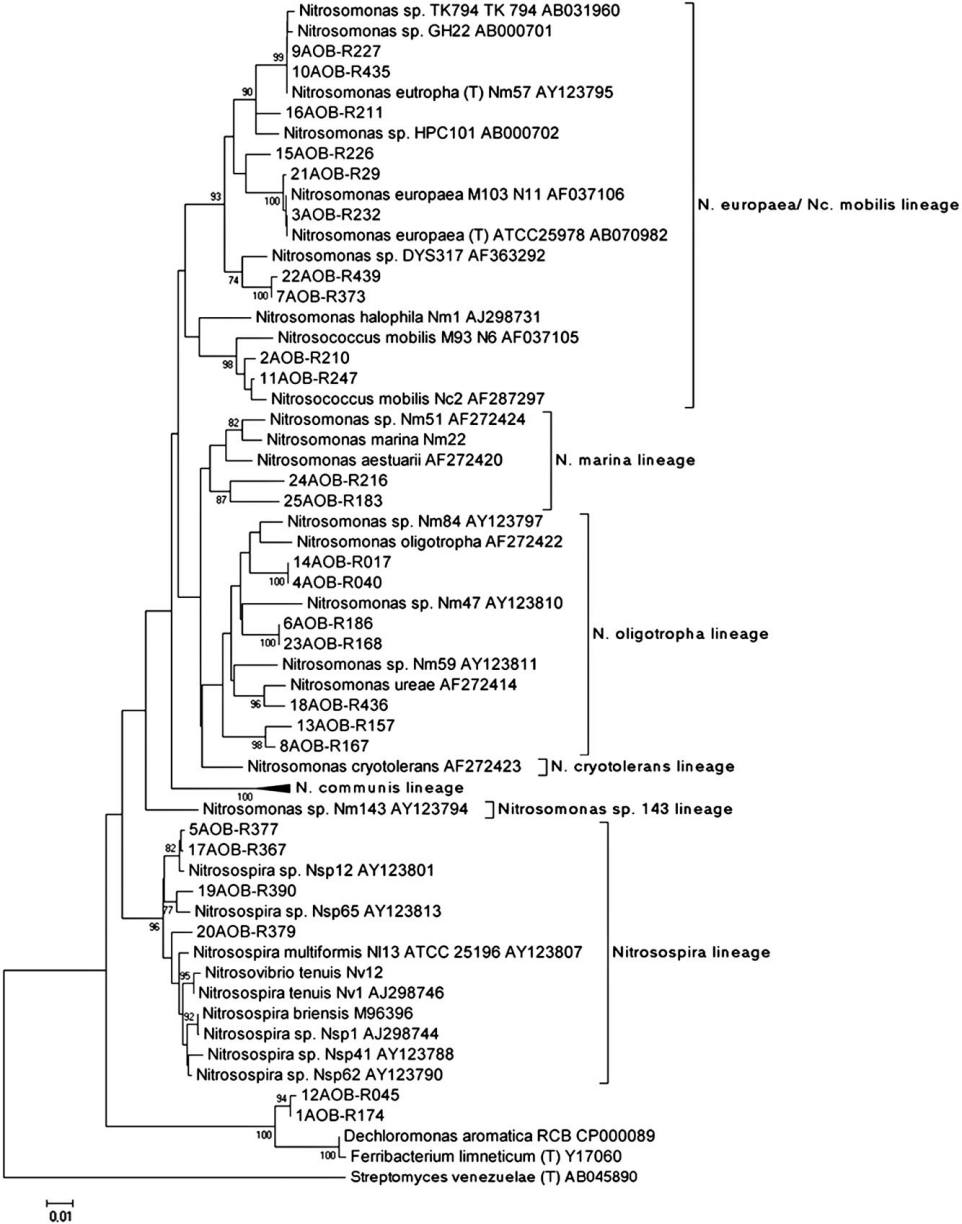
Phylogenetic tree of the AOB 16S rRNA gene sequences of the representative OTUs and their phylogenetic relatives constructed with mega5. The tree is based on minimum evolution method.

More detailed AOB community analyses were conducted on samples collected from the four reactors at the end of the experiment (day 73) by constructing AOB 16S rRNA gene clone libraries. The 169 partial 16S rRNA gene sequences were grouped in 25 operational taxonomic units (OTUs), which were distributed in the reactors as reported in Fig. 5. Representative sequences of each OTU were analysed to identify the most closely related sequences in the GenBank database (Table 2), and phylogenetic analysis was conducted (Fig. 6). Of the 25 OTUs, 22 showed between 96% and 100% identity to AOB 16S rRNA sequences present in the database, one had 95% of identity with *Nitrosomonas europaea*, and two were associated with non-AOB. Most of the sequences (80%) were closely related to sequences from members of the genus *Nitrosomonas*, while the rest were associated with *Nitrosospira* spp.. In R1-L_N_H_O_, members of *Nitrosomonas oligotropha* were dominant, though they coexisted with *Nitrosomonas ureae* and *Nitrosomonas* sp. Nm51. The sequences recovered from R2-L_N_L_O_ were related mostly to *Nitrosococcus mobilis*, *Nitrosomonas eutropha*, and *N. europaea*, but a few sequences were related to *N. oligotropha* and *Nitrosomonas marina*. In R4-H_N_L_O_, an *N. eutropha*-like bacterium was the dominant AOB, but organisms related to *N. oligotropha*, *N. ureae*, *Nitrosospira* and *N. europaea* were also detected. In contrast with the other reactors, most of the sequences retrieved from R3-H_N_H_O_ were related to *Nitrosospira* spp., and only one clone was associated with the *N. oligotropha* lineage.

**Table 2:** Closest matches (BLASTN) of the sequences of the representative OTUs retrieved from the AOB clone libraries. The query coverage (QC), max identity (MI) and reference are also reported

OTU	QC (%)	MI (%)	Accession	Description
1AOB-R174	100	99	EU924130	Uncultured *Dechloromonas* sp., clone AOB_B12 16S ribosomal RNA gene
2AOB-R210	100	99	AF527019	Uncultured *Nitrosomonas* sp., 36Fb 16S ribosomal RNA gene
3AOB-R232	100	99	AL954747	*N. europaea* ATCC 19718, complete genome
4AOB-R040	100	99	EU285320	Uncultured *Nitrosomonas* sp., clone AOB17 16S ribosomal RNA gene
5AOB-R377	99	99	EU285326	Uncultured *Nitrosospira* sp., clone AOB23 16S ribosomal RNA gene
6AOB-R186	100	98	FM201082	Uncultured *Nitrosomonas* sp., partial 16S rRNA gene, clone MBR-8_LF_BF68
7AOB-R373	98	96	GQ451713	*N. europaea* strain ATCC 25978 16S ribosomal RNA gene
8AOB-R167	100	97	AF527014	Uncultured *Nitrosomonas* sp, 21Fb 16S ribosomal RNA gene
9AOB-R227	100	99	CP000450	*Nitrosomonas eutropha* C91, complete genome
10AOB-R435	98	100	CP000450	*Nitrosomonas eutropha* C91, complete genome
11AOB-R247	98	99	AJ298728	*Nitrosococcus mobilis* partial 16S rRNA gene, isolate Nc2.
12AOB-R045	100	99	EU924131	Uncultured *Dechloromonas sp*., clone AOB_B3 16S ribosomal RNA gene
13AOB-R157	98	98	AY543074	Uncultured *Nitrosomonas* sp., clone 15BAFln1 16S ribosomal RNA gene
14AOB-R017	100	99	EU285320	Uncultured *Nitrosomonas* sp., clone AOB17 16S ribosomal RNA gene
15AOB-R226	100	98	AF386750	*Nitrosomonas* sp. R5c88 16S ribosomal RNA gene, partial sequence.
16AOB-R211	99	97	CP000450	*Nitrosomonas eutropha* C91, complete genome
17AOB-R367	100	99	EU285326	Uncultured *Nitrosospira* sp., clone AOB23 16S ribosomal RNA gene
18AOB-R436	98	99	DQ002458	Uncultured *Nitrosomonas* sp., clone EZS-3 16S ribosomal RNA gene
19AOB-R390	100	99	AY138531	*Nitrosospira* sp., DNB_E1 16S ribosomal RNA gene
20AOB-R379	100	98	X84661	*Nitrosospira* sp., 16S rRNA gene, isolate T7.
21AOB-R29	98	99	AL954747	*N. europaea* ATCC 19718, complete genome
22AOB-R439	100	95	AL954747	*N. europaea* ATCC 19718, complete genome
23AOB-R168	100	97	EF016119	*N. oligotropha* isolate AS1 16S ribosomal RNA gene
24AOB-R216	100	97	AF386753	*Nitrosomonas* sp. R7c140 16S ribosomal RNA gene, partial sequence.
25AOB-R183	100	98	AF527021	Uncultured *Nitrosomonas* sp., 11Fb 16S ribosomal RNA gene

### Species richness

The number of OTUs can be used as an estimation of species richness. The 16S rRNA gene clone library data showed that the AOB communities selected in each reactor were made up of different numbers of species (S) as reported in Table 3. However, the observed diversity resulted slightly lower than the estimated one by non parametric diversity indices, Chao1 and ACE, as the rarefaction curves revealed that the AOB communities were under sampled in the AOB clone libraries, though the rates of OTU accumulation were beginning to decelerate (Fig. S3).

**Table 3:** Observed (AOB 16S rRNA gene clone libraries and DGGE) and estimated AOB species richness

	Reactors			
	R1-L_N_H_O_	R2-L_N_L_O_	R3-H_N_H_O_	R4-H_N_L_O_
Number of OTUs^a^	8	11	6	7
Chao1 (standard error)	9 (3.4)	14 (11.7)	6.5 (3.7)	7.3 (1.9)
ACE (standard error)^b^	10.7 (1.5)	15.14 (2)	7.6 (1.2)	8.4 (1.4)
Number of bands (standard deviation)^c^	9 (1.5)	7 (0.8)	11.75 (0.5)	5.5 (0.6)

a Number of OTUs observed in the AOB 16S rRNA gene clone libraries without considering the non-AOB OTUs.

b Abundance-based coverage estimation (ACE).

c Mean values of the number of bands detected in the DGGE profiles of the samples collected the last four sampling days (46, 55, 64, 73) in each reactor.

### AOB community composition and RRT

**Fig 7:**
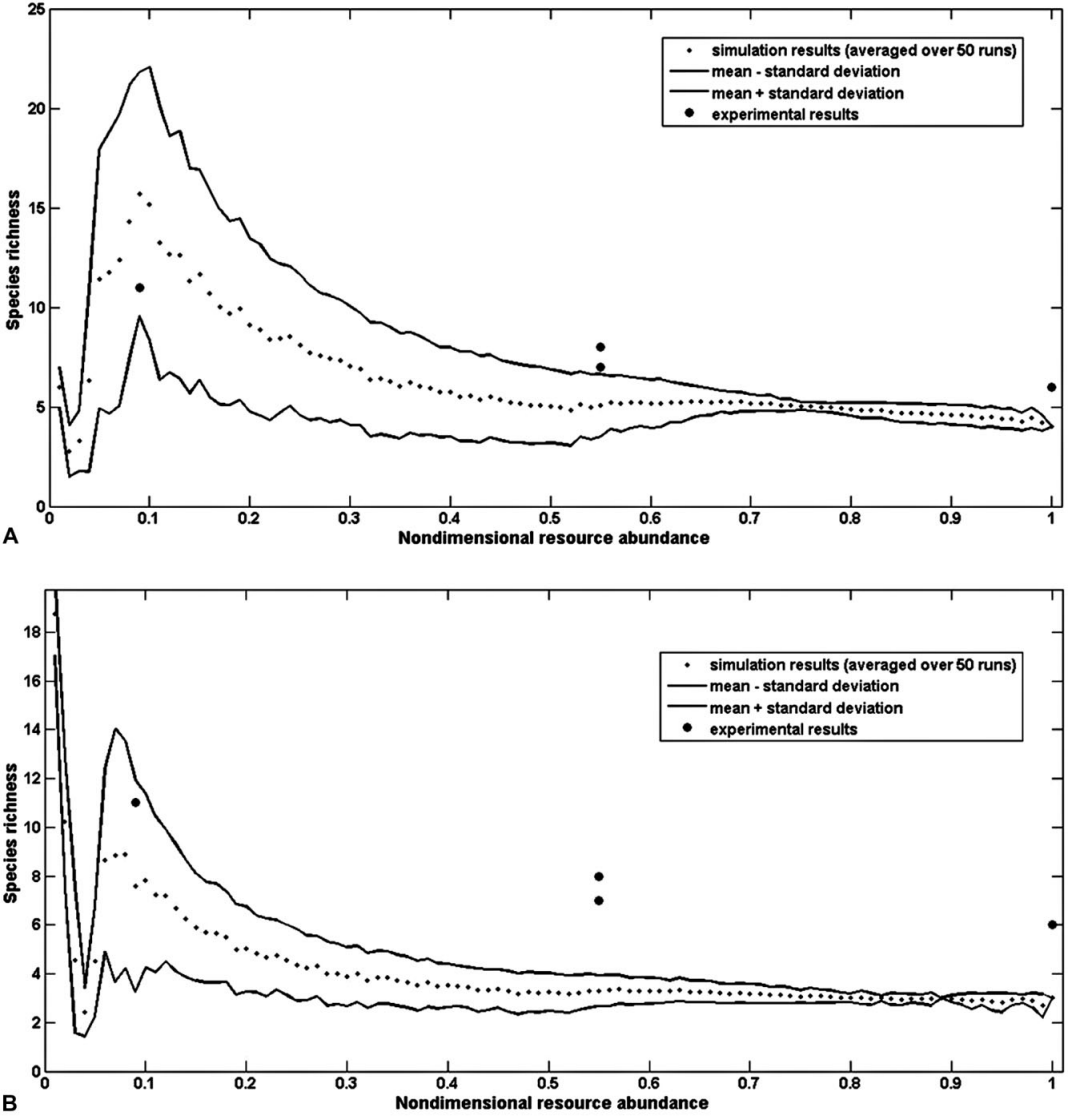
Comparison between empirical and theoretical results when the variation of oxygen consumption (A) and the minimum microhabitat containing all the species (B) are taken into account.

The AOB species richness values (as defined by OTUs) observed in the reactors were compared with the simulations of the RRT. In the first set of simulations, the activated sludge floc was considered as a heterogeneous environment, where the ammonia and oxygen levels vary with the depth of the floc. The variation in ammonia and oxygen through the floc was modelled by taking into consideration the diffusion–consumption of the two resources. The mathematical computation of the diffusion–consumption of the oxygen revealed a non-dimensional variation (σ) of oxygen equal to 0.014, whereas the variation of the ammonia concentration in the floc was negligible (σ = 0). Tilman (1982) defined heterogeneity in the environment as the 0.99 probability contour of a bivariate normal distribution of resource concentrations, which is obtained at 2.58·σ and by assuming that the two resources are independent (r = 0) and have the same variance (σ = σ_1_ = σ_2_). The simulated species richness-resource abundance curve is humped (Fig. 7A), suggesting that habitats with very low resource levels cannot support large numbers of species, but in general low resources lead to maximal diversity, while a high level of resources leads to a decrease in the number of species. The standard deviation of the species richness is high in the same gradient of resources, suggesting that it is intrinsically more difficult to predict AOB diversity at low resource levels (Fig. 7B). The experimental data (closed dots in Fig. 7A) were very close to the simulated range.

The second set of simulations was run taking into account the minimum microhabitat containing all the species. Overall, the trend of the resulting species richness-resource gradient curve and relative standard deviation concurred with the initial simulation, though the predicted AOB species richness was generally lower (Fig. 7B).

## Discussion

In this study, we successfully manipulated the AOB community structure of lab-scale nitrifying systems according to the two growing limiting resources supplied. Different amounts of oxygen and ammonia selected for AOB communities different in richness and composition as shown by DGGE and clone libraries analyses of AOB 16S rRNA genes. This suggests that the principles of RRT have the potential to help predicting and possibly managing some characteristics of microbial communities in WWTPs, in particular, and open microbial systems, in general. The qualitative agreement with the simulation was also gratifying in what was a simple first attempt with parameters taken from the literature.

We would however, offer substantial *caveats*. First, our reactors were not replicated. Nevertheless, in a previous study where the same bioreactors were operated in duplicate but with different operating conditions from this study, we demonstrated that AOB communities are highly replicable with a coefficient of variation between 0.04 and 0.1 (Bellucci *et al*., 2011). Though replication would have been ideal, and it is proposed for a future study, a statistically robust replication of the pattern would not prove the underlying mechanism envisaged in RRT. That would require us to demonstrate that the kinetic parameters of the microbes were consistent with diversities observed.

This in turn would require a robust calibration of our preliminary simulation. This is possible but challenging. However, even with a perfect calibration of the model, accurate prediction of diversity would be difficult as the computational simulations in our study suggest that the diversity is intrinsically more variable in habitats supplied with low amounts of resources than in enriched environments. This, together with analogous findings in a recent study, modelling the outcome of competition between nine phytoplankton species for nitrogen, phosphorus and light (Brauer *et al*., 2012), might explain why several experimental works failed to fully corroborate the predictions of RRT (Miller *et al*., 2005). This *caveat* should be carefully considered when applying RRT in the design of WWTPs.

The modest discrepancy between experimental and predicted data is not surprising. The model used in this study requires large number of unknown parameters, and, therefore, provides only a crude estimation of the diversity. The number of species detected by the model is affected by the size of the microhabitat given by the variance of the resources through the activated floc. It would have been possible, but not meaningful, to adapt the literature values used to fit our experimental findings.

Nevertheless, the low concentration of resources in R2-L_N_L_O_ selected for a community with the highest number of species, while the AOB community in the reactor with high oxygen and N input (R3-H_N_H_O_) was the least diverse, in agreement with the predictions of RRT. The low diversity retrieved from R3-H_N_H_O_, which was supplied with the highest amount of resources suggests that the ‘paradox of enrichment’ (Rosenzweig, 1971) can be found in engineered systems, at least in autotrophs.

In so far as we can tell, the presence of the taxa in the systems is consistent with the physiologies associated with these phylotypes. For example, our work is consistent with an earlier study in chemostats (Park and Noguera, 2007) as the low oxygen and ammonia conditions in R2-L_N_L_O_ favoured the parallel growth of members of the *N. oligotropha* lineage and *N*. *europaea*. Apparently, the contrasting affinity for oxygen and ammonia of these AOB represents a strategy for their coexistence in the system (Park and Noguera, 2007). The ability of *N. europaea* to use nitrite as electron acceptor (Chain *et al*., 2003) could also be a selective advantage in system with high nitrite levels, such as R2-L_N_L_O_. On the other hand, the high concentration of ammonia apparently promoted the establishment of an AOB community dominated by *Nitrosospira* and *N. eutropha* in R3-H_N_H_O_ and R4-H_N_L_O_, respectively. From an ecological prospective, our findings run counter to the consensus that *Nitrosospira* spp. are typical K-strategists with high affinity for ammonia and low growth rate compared with *N. europaea* spp., which are considered to be r-strategists (Andrews and Harris, 1986). However, this definition resulted from evidence of *Nitrosomonas* spp. rather than *Nitrosospira* in ammonium-rich activated sludge and biofilm reactors in previous studies where the pH was maintained near neutral values (Mobarry *et al*., 1996; Schramm *et al*., 1996; 1999; 2000; Terada *et al*., 2013). In our experiment the pH was not adjusted, and it decreased to ∼ 6 in R3-H_N_H_O_. Probably, the low pH, together with the high ammonia concentration of the influent, selected for *Nitrosospira* sp. as its capacity for urease production between pH 5 and 6 (Koops *et al*., 2003) provides a competitive advantage in such conditions. One the other hand, *N. eutropha*-like AOB are often found in the SHARON (Stable High rate Ammonia Removal Over Nitrite) processes in which high ammonia loads are imposed to promote nitrite accumulation (Logemann *et al*., 1998; van Dongen *et al*., 2000). *Nitrosomonas eutropha* can tolerate high concentrations of ammonium (600 mM) and compete with other AOB in systems where competition for oxygen with heterotrophic organisms is acute; thanks to the presence of a *nirK* gene in the genome (Stein *et al*., 2007). It could be speculated that the ammonia oxidizing archaea (AOA), which possess the *amoA* gene encoding for the ammonia monooxygenase (Schleper *et al*., 2005), might have contributed to the performance of our bioreactors, as they have been already found in natural and engineered systems with low DO concentrations (Park *et al*., 2006; Gómez-Silván *et al*., 2009; Zhang *et al*., 2009; Pitcher *et al*., 2011). However, AOA were not detected in our systems by polymerase chain reaction (PCR; results not shown); presumably, their growth and activity could not be sustained in our bioreactors that were operated with relatively high ammonia loading and short retention time (Hatzenpichler, 2012). This hypothesis can be confirmed only when the mechanisms selecting for AOA, and their real role, in activated sludge are better understood.

Overall, deterministic factors appeared to have a major role in shaping the AOB community of the reactors. Shifts in the community occurred as a function of the ammonia and oxygen supplied to the systems as most of the AOB community DGGE profiles taken from the same reactor formed distinct groups in cluster analysis (Fig. 3). This is in agreement with previous studies, suggesting that an AOB community with particular ecological requirements emerged from a common pool (the seed) and became dominant over time (Juretschko *et al*., 1998; Figuerola and Erijman, 2007; Ayarza *et al*., 2010; Bellucci *et al*., 2011; 2013; Beneduce *et al*., 2014). Oxygen exerted a stronger effect than ammonia on the selection of the AOB community as only the DGGE patterns of the samples collected after 28 days in R2-L_N_L_O_ and R4-H_N_L_O_ were significantly dissimilar to the original common pool of bacteria. Therefore, as Park and Noguera (2004; 2007) have previously reported, the oxygen supplied in R2-L_N_L_O_ and R4-H_N_L_O_ dictated the AOB community structure. The identification of the operational conditions required to establish the AOB species that can function efficiently in low oxygen environments is fundamental for the development of less costly WWTPs.

In conclusion, if we could find an authoritative and reliable method for calibrating the model, the RRT approach would contribute to the management of WWTPs and to the refinement of ecological theory. However, even with perfect calibration, at some resource ratios the predictions may be more variable than previously recognized.

## Experimental procedures

### Experimental set-up

#### Bench scale reactors

Four continuous flow lab-scale reactors were operated in parallel for 73 days. The reactors consisted of glass cylinders with a funnel bottom in which a porous glass grid was placed in the centre. The systems were inoculated (day 1) with 3 liters of return activated sludge from a Municipal Wastewater Plant in Spennymoor (County Durham, UK, 54° 42′ 0″ N, 1° 35′ 24″ W), and the liquor was constantly mixed by a stirrer (∼ 100 rpm). The reactors were set up using a 2 × 2 factorial design; the two factors considered were the percentage of oxygen supply and the inorganic ammonia concentration in the influent (Table 1). Two of the reactors were supplied with air (R1-L_N_H_O_ and R3-H_N_H_O_), while the other two systems were supplied with a gas mixture containing 2% of oxygen and 98% of nitrogen (R2-L_N_L_O_ and R4-H_N_L_O_). The gas flow rate was fixed at 0.2 l/min. Synthetic wastewater kept at 4°C was continuously pumped into the reactors at a fixed solid and hydraulic retention time (SRT and HRT) of 5 days. Synthetic wastewater consisted of either 66 mg/l (NH_4_)_2_SO_4_ in R1-L_N_H_O_ and R2-L_N_L_O_ or 1322 mg/L of (NH_4_)_2_SO_4_ R3-H_N_H_O_ and R4-H_N_L_O_, 320 mg/l peptone, 190 mg/l meat extract, 30 mg/l yeast extract, 30 mg/l urea, 28 mg/l K_2_HPO_4_, 2 mg/l CaCl_2_·2H_2_O and 2 mg/l of MgSO_4_·7H_2_O. The media were autoclaved (120°C for 20 min) prior to use and, after autoclaving, 1 ml/l of trace element solution (0.75 g/l FeCl_3_●6H_2_O, 0.075 g/l H_3_BO_3_, 0.015 g/l CuSO_4_●5H_2_O, 0.09 g/l KI, 0.06 g/l MnCl_2_●4H_2_O, 0.03 g/l NaMoO_4_●2H_2_O, 0.06 g/l ZnSO_4_●7H_2_O, 0.075 g/l CoCl_2_●6H_2_O, 0.5 g/l Ethylenediaminetetraacetic acid (EDTA) and 1 ml/l concentrated hydrochloric acid) and 0.7 mg/l NaHCO_3_ (Knapp and Graham, 2007) were added. Such solutions yielded different Total Kjeldahl Nitrogen (TKN), NH_4_^+^-N and COD concentrations, which are reported in Table 1. Ideally, replicate reactors could have been used, but we showed that performance and community structure was highly replicable in a previous study using identical reactors (Bellucci *et al*., 2011). Samples (200 ml) from each reactor and the feed synthetic wastewaters were collected every 3 days for chemical, physical and microbial community analyses.

#### Physical and chemical analyses

The dissolved oxygen concentration, temperature and pH were constantly monitored with specific probes (Broadley Technologies, UK). Nitrification performance and COD removal were assessed by analysing NH_4_^+^-N, NO_2_^−^-N, NO_3_^−^-N and COD levels in the bulk solution over time. Ammonium (NH_4_^+^-N) and COD concentrations were determined using the Ammonium Cell Test Kit and COD Cell Test (MERCK KGaA, Germany), respectively, according to the manufacturer's instructions. Measured were NO_2_^−^ and NO_3_^−^ by ion chromatography (DW-100 Ion Chromatography, Dionex, Sunnyvale, CA., USA); the system had an IonPac AS14A Analytical column, flow rate equal to 1 ml/min, the eluent was a 8.0 mM Na_2_CO_3_/1.0 mM NaHCO_3_ solution, and the injection loop was 25 μl. Free ammonia and free nitrous acid were calculated in function of the experimental values of total ammonia and nitrite concentrations, pH and temperature using the equation described by Anthonisen and colleagues (1976) and reported in the Supporting Information. Total suspended solid (TSS) and VSS, as well as the TKN in the medium supplied to the systems, were evaluated according to standard methods (APHA, 1998).

#### DNA extraction and quantitative PCR

DNA was extracted from the mixed liquor (250 μL from the four reactors every 9 days starting from day 1 to day 73) and stored at −20°C until further analyses. After mechanical cell lysis using Lysing Matrix E (MP Biomedicals, Solon, USA) in a Ribolyser (Hybaid, UK), DNA extraction was performed using the FastDNA SPIN kit For Soil (MP Biomedicals, Solon, USA) in accordance with manufacturer's instructions.

The abundance of AOB in the mixed liquor was evaluated by primers CTO 189fA/B (5′-GGAGRAAAGCAGGGGATCG-3′), CTO189fC (5′-GGAGGAAAGTAGGGGATCG-3′) (Kowalchuk *et al*., 1997) and RT1r (5′-CGTCCTCTCAGACCARCTACTG-3′) (Hermansson and Lindgren, 2001). The quantitative PCR (qPCR) reactions were performed in a BioRad iCycler equipped with an iCycler iQ fluorescence detector and associated software (BioRad Version 2.3). The 15 μl PCR reaction mixture contained 2 μl of DNA template, 1 μl of primer mixture (7.5 pmol each) and 12 μl of PCR Precision Mastermix PCR reagent (Primer Design, UK). For fluorescence detection, SYBR green I (10,000 x, Sigma, UK) was first diluted 1/100 in sterile and autoclaved molecular water, and such solution was added to the PCR reaction mixture as 1% of the total volume of the reaction mix (vol/vol). The thermal cycling was carried out as previously reported by Hermansson and Lindgren (2001). The 16S rRNA gene copy numbers were converted to equivalent cell numbers, assuming that one rRNA operon exists per AOB cell (Klappenbach *et al*., 2001).

#### Nested PCR and DGGE

A nested PCR approach, followed by DGGE was performed to monitor AOB population dynamics. In the first PCR, a 465 bp fragment of the 16S rRNA gene of AOB was amplified using CTO189F (5′-GAGRAAAGYAGGGGATCG-3′) and CTO654R (5′-CTAGCYTTGTAGTTTCAAACGC-3′) (Kowalchuk *et al*., 1997). The PCR products from this reaction were used as a template for a second PCR reaction with primer 3 (5′-CGCCCGCCGCGCGCGGCGGGCGGGGCGGGGGCACGGGGGGCCTACGGGAGGCAGCAG-3′) and primer 2 (5′-ATTACCGCGGCTGCTGG-3′) (Muyzer *et al*., 1993). All PCRs were carried out in 25 μl reactions containing 23.5 μl of PCR buffer (MegaMix-BLUE, Microzone, UK), 0.5 μl of each primer (10 pmol) and 0.5 μl of DNA template. The PCR conditions for the CTO primer set and primer 3 and primer 2 set were previously described (Muyzer *et al*., 1993; Kowalchuk *et al*., 1997), and all the PCR reactions were performed using a Px2 Thermal Cycler (Thermo Hybaid, Hybaid, UK).

Nested PCR amplified fragments were separated using a D-Code DGGE system (Bio-Rad Laboratories, UK) as described previously (Bellucci and Curtis, 2011; Bellucci *et al*., 2011, Bellucci *et al*., 2013, Beneduce *et al*., 2014). The gels were processed using Bionumerics 4.0 (Applied Maths BVBA, Saint-Martens-Latem, Belgium). Gels were normalized using a reference marker that was loaded into the polyacrilamide gels every eight samples and co-migrated with them. This approach allows comparing community profiles within the same gel and among differing gels. Pairwise similarities between DGGE profiles were calculated with Raup and Crick coefficients for cluster (single linkage clustering) and moving window analyses. The Raup and Crick similarity index (S_RC_) is defined as the probability that the expected similarity (randomized data) would be greater than or equal to the observed similarity (Raup and Crick, 1979). Similarity values between 0.05 and 0.95 are indicative of random occurrence of the same organism (DGGE band) in two samples, whereas S_RC_ above 0.95 and below 0.05 indicate significant similarity and significant dissimilarity, respectively. The algorithm assumes the taxa are equally likely to be selected. Cluster analysis was conducted using PAST (Hammer *et al*., 2001). In the moving windows analysis the similarity between DGGE profiles of a given day with the previous sampling day was calculated for each configuration.

The number of bands detected in the DGGE profiles could be considered as a rough estimation of AOB diversity, though it should be kept in mind that two amplicons differing in sequences may migrate together, and the same species might contain several copies of the same gene that differ slightly in sequence producing several bands in the DGGE. Nevertheless, the number of bands in the profiles of the samples collected the last four sampling days (46, 55, 64, 73) were counted and compared to confirm further that the AOB community richness was stable at the end of the reactor operation.

#### Clone library preparation

To evaluate and compare the AOB community structure selected in the four reactor configurations after 73 days of operation, gene clone libraries were constructed from AOB partial 16S rRNA genes amplified from samples collected from each reactor at the end of the experiment. Polymerase chain reaction products amplified with the CTO primer set were cloned with a TOPO TA cloning kit (Invitrogen, UK). Following PCR amplification, the correct size of the fragment was checked by electrophoresis in a 1.5% agarose gel run in 1 × TAE buffer stained with ethidium bromide. The bands were excised and purified with a Qiaquick PCR Gel Extraction Kit (QIAGEN, UK). The PCR products and the vector provided by the kit were ligated and used to transform OneShot competent cells according to the manufacturer's instructions (Invitrogen, UK). Clones (37–42) were randomly picked and transferred to a 30 μl PCR mixture containing the primers T3 (5′-ATTAACCCTCACTAAAGGGA-3′) and T7 (5′-TAATACGACTCACTATAGGG-3′). Polymerase chain reaction products of the correct size were prepared for sequencing using ExoSAP-IT (GE Healthcare, UK), and the sequences of the partial 16S rRNA gene fragment (ca 465 pb) were determined by Genevision, UK.

The sequences obtained were aligned using ClustalX v1.83 (Thompson *et al*., 1997), and vector and primer sequences were removed. The triggered sequences with > 97% identity were grouped by FastGroupII (Yu et al., 2006) into OTUs and the representative sequences of each group were analysed using BLASTn. The representative OTUs were also checked for putative chimeras with the online software Bellerophon (Huber *et al*., 2004) using a window size of 200 pb. Molecular Evolutionary Genetics Analysis (MEGA) version 5 (Tamura *et al*., 2011) was used to conduct phylogenetic analysis. The evolutionary history was inferred using the Minimum Evolution (ME) method (Rzhetsky and Nei, 1992) using the OTUs found in this study and selected AOB and *Ferribacterium* sequences (62 in total) for which 16S rRNA sequences longer than 1200 bp are available in the database Ribosomal Database Project (Maidak *et al*., 1999). The bootstrap consensus tree was inferred from 1000 replicates (Felsenstein, 1985). Branches corresponding to partitions reproduced in less than 50% bootstrap replicates were collapsed. The evolutionary distances were computed using the maximum composite likelihood method (Tamura *et al*., 2004) and are in the units of the number of base substitutions per site. The ME tree was searched using the close-neighbour-interchange algorithm (Nei and Kumar, 2000) at a search level of 1. The Neighbour-joining algorithm (Saitou and Nei, 1987) was used to generate the initial tree. On the basis of the OTUs detected in the AOB clone libraries, the AOB diversity was assessed as observed richness and non-parametric estimates of AOB richness, Chao1 and ACE, with the software r (R Development Core Team, 2011) using the package Vegan and the function estimator (Oksanen *et al*., 2013). The AOB observed and estimated richness was compared with the rarefied one by building individual rarefaction curve with PAST (Hammer *et al*., 2001).

#### Nucleotide sequence accession numbers

The sequences determined in this study have been deposited in the GenBank database under accession number KC346976-KC347000.

## Model description

Resource-ratio theory states that the quantity of the growth limiting resources available in a heterogeneous environment determines the species richness of a biological community (Tilman, 1982). The underpinning theory is provided in the Supporting Information, including a numerical model developed to simulate the theory. Specifically, an AOB community of 23 species was mathematically simulated based on the competition for oxygen and ammonia as primary resources using the RRT model. The model assumes microhabitats are best represented as explicit circles (with defined radius) around resource-intersection points associated with mean values of the two resources (see Fig. S1 and SI). The radius of each microhabitat was defined (i) by inferring the diffusion and consumption of a given resource and (ii) by considering the minimum resource variance suitable to comprise all the potential species. Model assumptions are provided in the Supporting Information (Table S1). For each given radius, 50 replicates were generated. The averages of the species richness and the relative standard deviations were then plotted against the resource.

## Supporting Information

**Fig. S1.** Competition among four species (A–D) for two resources (Resource 1 and Resource 2). The grey dash line is the line on which the Zero Net Growth Isocline (ZNGIs) are placed randomly. The ZNGIs and the consumption vectors are the continuous and dotted lines, respectively. The circles represent the microhabitats given by the 0.99 probability contour of the bivariate distribution. The number of species coexisting in each microhabitat is also specified near each circle. The grey stars are the cross points of two ZNGIs from which the consumption vectors are generated. The black dots define the diameter of the circle comprising all the potential species. **Fig. S2.** COD removal (A), pH (B), concentrations of FA (C) and FNA (D) observed in the four bioreactors over time. **Fig. S3.** Rarefaction curves based on the OTUs observed in the AOB clone libraries. **Table S1.** Parameters used to simulate the AOB diversity.
