# Supplementary material for: A preliminary and qualitative study of resource ratio theory to nitrifying lab-scale bioreactors
Source: Microb Biotechnol. 2015 Apr 15;8(3):590–603. doi: 10.1111/1751-7915.12284 (PMC4408191; doi:10.1111/1751-7915.12284)
Supplement: Supplementary file 1 [file mbt20008-0590-sd1.docx]

Supporting Information

A PRELIMINARY AND QUALITATIVE STUDY OF RESOURCE RATIO THEORY TO NITRIFYING LAB SCALE BIOREACTORS

Micol Bellucci^1,2*^, Irina D. Ofiţeru^3,4^, Luciano Beneduce^2^, David W. Graham^1^, Ian M. Head^1^ and Thomas P. Curtis^1^

^1^ School of Civil Engineering and Geosciences, Newcastle University, Newcastle upon Tyne, NE1 7RU, United Kingdom.

^2^ Dipartimento di Scienze Agrarie, Alimentari ed Ambientali, Università di Foggia, via Napoli 25, 71121, Foggia, Italy.

*^3^ School of Chemical Engineering and Advanced Materials, Newcastle University, Newcastle upon Tyne, NE1 7RU, United Kingdom.*

^4^ Chemical Engineering Department, University Politehnica of Bucharest, 011061 Polizu 1-7, Bucharest, Romania.

* Corresponding author:

School of Civil Engineering and Geosciences, Newcastle University, Newcastle upon Tyne, NE1 7RU, United Kingdom.

Phone: +44 (0)191 208 6323

Fax: +44 (0)191 208 6502

Present address

Dipartimento di Scienze Agrarie, Alimentari ed Ambientali, Università di Foggia, via Napoli 25, 71121, Foggia, Italy.

E-mail: [micol.bellucci@gmail.com](mailto:micol.bellucci@gmail.com)

Running title: RRT in nitrifying lab scale bioreactors

**Resource Ratio Theory Model Description**

Background: RRT states that the quantity of the growth limiting resources available in a heterogeneous environment determines the species richness of a biological community (Tilman, 1982). In homogeneous habitats, the competition for two growth limiting resources (R_1_ and R_2_) among more than one species is described in Figure S1. In this plot the Zero Net Growth Isoclines, or ZNGI, represent the amount of the two growth limiting resources that must be available in the system such that growth is equal to the mortality rate. Different species have different ZNGIs. In order to maintain an equilibrium population, the resource consumption rate must balance the resource supply rate. This equilibrium point is plotted as consumption vectors (C_v_). The C_v_, along with the ZNGIs, defines different regions (homogenous habitats) in which either two species coexist or one of the two becomes dominant. To apply those predictions to heterogeneous habitats, microhabitats are included in the plots. Those are graphically expressed as circles (Figure S1) given by the 0.99 probability contour of a bivariate distribution calculated by the mean and variance of the two resources in each habitat. The species richness and composition in each microhabitat is inferred by evaluating the diversity of the differing homogeneous regions overlapping the circles.

Here we simulated an AOB community of 23 species to assess competition for oxygen and ammonia as casted for the RRT model. Tilman’s model was recast by making the following assumptions:

- The activated sludge floc is a heterogeneous environment, and offers more than one potential microhabitat, as ammonia and oxygen vary as a function of depth in activated sludge flocs (Li and Bishop, 2004).
- 23 AOB species (total number of OTUs retrieved in the AOB 16S rRNA gene clone libraries) compete for oxygen and ammonia.
- In the resource space framework space, the ammonia concentration, resource1, ranges between 0 and 280 mg/l (the latter being the maximum ammonia concentration in the inlet), whereas the oxygen supplied, resources2, varies between 0 and 21%. The amount of the two resources is normalized in order to have a range between 0-1.
- The ZNGIs and the consumption vectors are chosen randomly as they depend on the species’ growth and mortality rate, as well as on the affinities for the two resources. In this experimental set it was not possible to define these parameters for each species. Consequently, the ZNGIs and consumption vectors were placed in the framework as follows:

1. An incline line passing through the minimum amount of the two resources supplied to the bioreactors (24 mg/l of ammonia and 2 % of oxygen) was defined in the simulation space (grey dash line in Figure S1). We have assumed that no species could survive under those values.
2. On the incline line we have randomly defined 23 points, from which the ZNGIs had been originated.

3) The point where the ZNGIs cross (stars in Figure S1) is defined as a two species equilibrium point, in which the two species coexist. Consumption vectors of the two species were derived with a random slope from each crossing point.

- The microhabitats were represented as circles (500) and placed randomly in the resource space by generating their centre, which corresponds to the mean values of the two resources.
- The size of the microhabitat (the radius of the circle) was defined (i) by inferring the diffusion and consumption of a given resource and (ii) by considering the minimum resource variance suitable to comprise all the potential species.

For each given radius 50 replicates were generated. The averages of the species richness and the relative standard deviations were then plotted against the resource gradient (as (resource1 + resource2)/2) and compared with the experimental data.

Calculating the circle radius using diffusion and consumption of resources: The gradient of the resources (i.e. ammonia or oxygen) through a fixed size floc was calculated using a diffusion-consumption dynamic mass balance equation (Levenspiel, 1999):

$\frac{\partial S_{i}}{\partial t}=D_{i}\frac{\partial^{2}S_{i}}{\partial x^{2}}-r_{i}$ (1)

where *S* is the *i*^th^ resource concentration, *D* is the diffusion coefficient for the resource *i*, *r* is the consumption rate, *t* represents the time, and *x* is the floc depth. We assumed that the floc has a diameter of 50 μm (radius equal 25 μm ) (Zartarian *et al.*, 1997; Zhang *et al.*, 1997). The consumption rate *r* was calculated using Monod kinetics for each of the two substrates.

To calculate the diffusion and consumption of the resources, equation (1) was transformed in a dimensionless form as follows:

$\frac{\partial S_{i}}{\partial t}=D_{i}\frac{\partial^{2}S_{i}}{\partial x^{2}}-\mu_{\max}\frac{S_{i}}{K_{i}+S_{i}}X\cdot Y_{S_{i}/X}$ (2)

by considering $\tau=\frac{t}{\bar{t}}$ , where $\bar{t}$is the SRT (3 day); $\chi=\frac{x}{R}$, where R is the radius of the activated sludge floc; , where *S_bulk_* is the concentration outside the activated sludge floc (mg/l) (maximum concentration of ammonia and oxygen in the bulk solution),

The equation (2) can thus be rearranged as:

$\frac{\partial\xi_{i}}{\partial\tau}=\frac{D_{i}\cdot\bar{t}}{R^{2}}\cdot\frac{\partial^{2}\xi_{i}}{\partial\chi^{2}}-\mu_{\max}\cdot\bar{t}\cdot Y_{S_{i}/X}\cdot\frac{X}{S_{bulk,i}}\cdot\frac{\xi_{i}}{\frac{K_{i}}{S_{bulk},i}+\xi_{i}}$ (3)

having the initial and boundary conditions:

$\left\{\begin{matrix} \begin{matrix} \chi=0\text{, }\forall\tau\text{, }\frac{\partial\xi_{i}}{\partial\chi}\text{=0 }\left(\text{finite substrate decrease}\right) \\ \chi=1,\text{ }\forall\tau\text{, }\xi_{i}=1\text{ }\left(\text{no external resistance for substrate}\right) \end{matrix} \\ \tau= 0\text{, 0}\leq\chi<1,\text{ }\xi_{i}=0\text{ }\left(\text{no initial substrate in the floc}\right) \end{matrix}\right.$ (4)

The parameters used to define the variation of the oxygen and ammonia through the activated sludge floc are reported in Table S1. To delineate the circles in the resource space framework, we assumed that the variation (σ) of the two resources within the activated sludge floc is the same (σ_1_=σ_2_). The 0.99 probability contour of the bivariate distribution is given by multiplying σ for 2.58 (Sokal and Rohlf, 1995).

Radius of the circle defined as minimum resource variance comprising all the potential species: Theoretically, there should be a microhabitat in which all the species coexist. This microhabitat can be defined as a circle that encompasses all the species. In order to define the radius of this circle, we outlined the circle that circumscribes the corner of the first and the last ZNGI present in the framework, which is the only region in the simulation space where all the species coexist.

**Determination of free ammonia and free nitrous acid**

Free ammonia (FA) and free nitrous acid (FNA) in the bioreactors were calculated in function of the experimental values of total ammonia and nitrite concentrations, pH and temperature using the following equation described by Anthonisen and colleagues (Anthonisen *et al.*, 1976):

$FA as \mathrm{NH}_{3}( \mathrm{mg}/L) =\frac{17}{14} \times\frac{total ammonia as N \left( \mathrm{mg}/L \right) \times{10}^{\mathrm{pH}}}{\left( {K_{b}}/{K_{w}} \right)+ {10}^{\mathrm{pH}}}$,

${{{in which K}_{b}}/{K_{w}}=e}^{{(6334}/{(273+T\left( ^{\circ}C \right))}}$

$FNA as \mathrm{HNO}_{2}( \mathrm{mg}/L)=\frac{46}{14} \times\frac{{\mathrm{NO}_{2}}^{-}-N as N \left( \mathrm{mg}/L \right)}{K_{a}+ {10}^{\mathrm{pH}}}$,

$${{in which K}_{a}=e}^{{(-2300}/{(273+T\left( ^{\circ}C \right))}}$$

where:

K_b_ = ionization constant of the ammonia equilibrium equation

K_w_ = ionization constant of water

K_a_ = ionization constant of the nitrous acid equilibrium equation

T= temperature (°C)

The results are summarized in Figure S2.

**Rarefaction curves**

Rarefaction curves based on the OTUs observed in the AOB clone libraries were constructed and reported in Figure S3.

**REFERENCES**

Anthonisen AC, Loehr RC, Prakasam TBS, Srinath EG (1976). Inhibition of nitrification by ammonia and nitrous acid. *Journal of the Water Pollution Control Federation* **48:** 835-852.

Levenspiel O (1999). *Chemical Reaction Engineering*, Third edition edn.

Li B, Bishop PL (2004). Micro-profiles of activated sludge floc determined using microelectrodes. *Water Research* **38:** 1248-1258.

Metcalf, Eddy (2003). *Wastewater Engineering: Treatment and Reuse*, 4th edition edn. The McGraw-Hill Companies, Inc.: New York.

Rittmann BE, McCarty PL (2001). *Environmental biotechnology: principles and applications*. McGraw-Hill Book Co: Singapore.

Sokal RR, Rohlf JF (1995). *Biometry : the principle and practice of statistics in biological research*, 3d ed. edn. W. H. Freeman and Company: New York.

Tilman D (1982). *Resource competition and community structure. Monographs in Population Biology*. Princeton University Press.

Zartarian F, Mustin C, Villemin G, Ait-Ettager T, Thill A, Bottero JY *et al* (1997). Three-Dimensional Modeling of an Activated Sludge Floc. *Langmuir* **13:** 35-40.

Zhang B, Yamamoto K, Ohgaki S, Kamiko N (1997). Floc size distribution and bacterial activities in membrane separation activated sludge processes for small-scale wastewater treatment/reclamation. *Water Science and Technology* **35:** 37-44.

Table S1 Parameters used to simulate AOB diversity

| Symbol | Name | Value and unit | References |
| --- | --- | --- | --- |
| D_O2_ | diffusion coefficient for oxygen | 2 x 10^-9^ m^2^/s, | (Levenspiel, 1999) |
| μ_max_ | maximum specific growth rate of the heterotrophs | 5 g VSS/ g VSS d | (Metcalf and Eddy, 2003); |
| K_s_ | affinity constant for oxygen for the heterotrophs | 1 mg/l | (Rittmann and McCarty, 2001) |
| Y | yield of heterotrophs | 0.40 g VSS/ g COD | (Metcalf and Eddy, 2003) |
| X | biomass of the heterotrophs in the reactors | 300 mg VSS /l. | This study |
| S*_bulk, O2_* | maximum concentration of DO in the reactors | 9.12 mg/l. | (Levenspiel, 1999) |
| D*_ammonia_* | diffusion coefficient for ammonia | 0.7 x 10^-9^ m^2^/s | (Levenspiel, 1999) |
| μmax_AOB | maximum specific growth rate of the AOB | 1.02 g VSS/ g VSS d | (Rittmann and McCarty, 2001) |
| K_n_ | saturation constant for ammonia | 1.50 mg/l | (Rittmann and McCarty, 2001) |
| Y | yield of ammonia consumption by AOB | 1/0.33 | (Rittmann and McCarty, 2001) |
| X | biomass of AOB in the reactors | 12 mg VSS /l * | This study |
| S*_bulk_*, _NH3_. | maximum concentration of ammonia in the reactors | 280 mg NH_4_^+^-N/l | This study |

*AOB comprise 4% of the total biomass


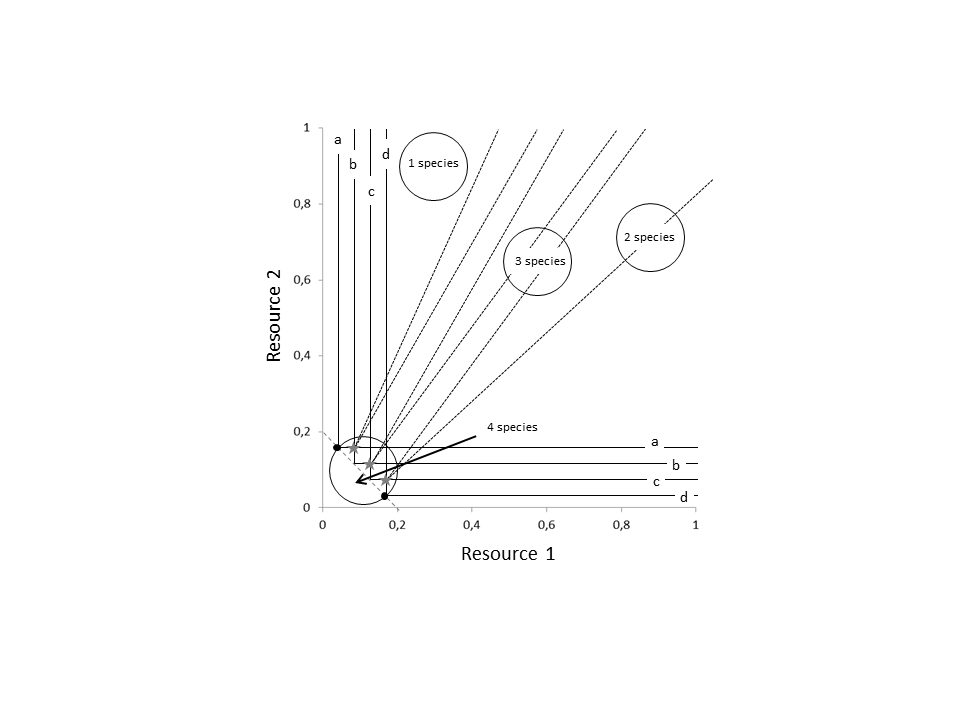


Fig. S1 Competition among four species (a-d) for two resources (Resource 1 and Resource 2). The grey dash line is the line on which the Zero Net Growth Isoclines (ZNGIs) are placed randomly. The ZNGIs and the consumption vectors are the continuous and dotted lines, respectively. The circles represent the microhabitats given by the 0.99 probability contour of the bivariate distribution. The number of species co-existing in each microhabitat is also specified near each circle. The grey stars are the cross points of two ZNGIs from which the consumption vectors are generated. The black dots define the diameter of the circle comprising all the potential species.


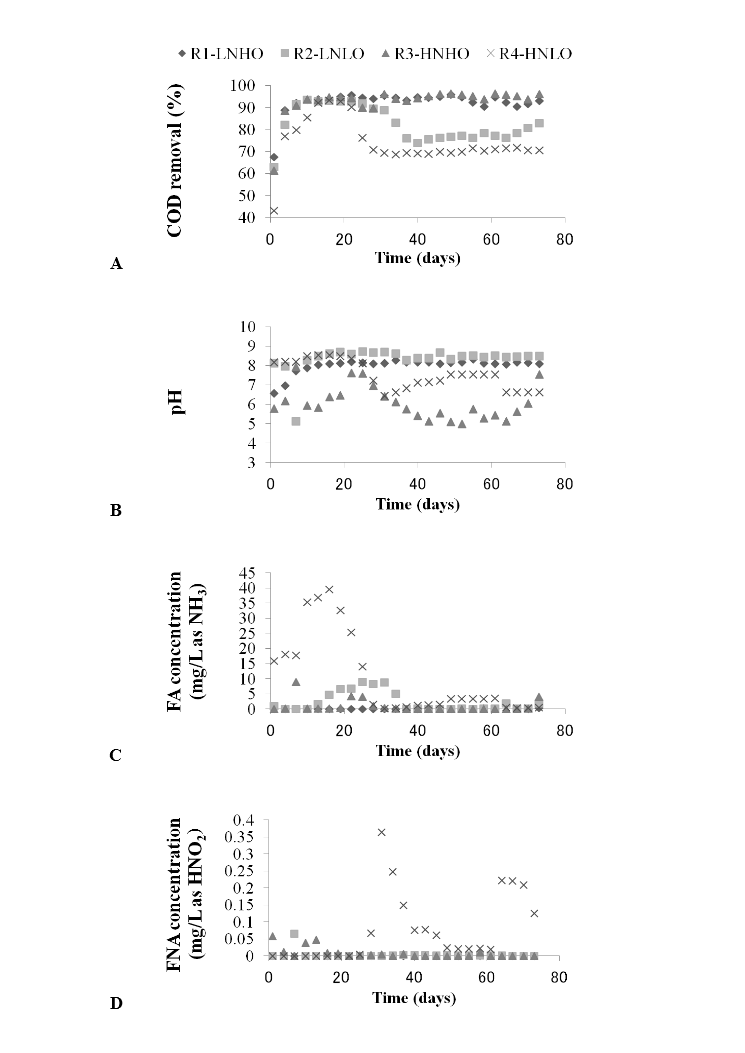


Fig. S2 COD removal (A), pH (B), concentrations of FA (C) and FNA (D) observed in the four bioreactors over time.


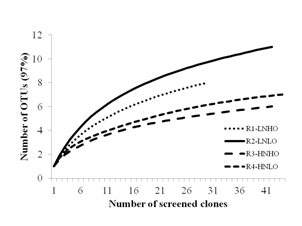


Fig. S3 Rarefaction curves based on the OTUs observed in the AOB clone libraries.
